# Supplementary material for: Mechanisms governing the pioneering and redistribution capabilities of the non-classical pioneer PU.1
Source: Nat Commun. 2020 Jan 21;11:402. doi: 10.1038/s41467-019-13960-2 (PMC6972792; doi:10.1038/s41467-019-13960-2)
Supplement: Supplementary file 7 — Source data [file 41467_2019_13960_MOESM7_ESM.zip › Source_Data/FigureS2/FigureS2B_MotifScanOutput/KmeansCluster5/homerResults/motif17.similar.html]

motif17

## Information for motif17

C
A
G
T
A
C
T
G
G
A
C
T
G
A
C
T
G
C
A
T
T
C
G
A
T
C
G
A
T
C
G
A
C
G
T
A
T
C
A
G
C
G
A
T
T
C
A
G
G
C
A
T
C
A
T
G
  
Reverse Opposite:  

G
T
A
C
C
G
T
A
A
G
T
C
G
C
T
A
A
G
T
C
G
C
A
T
A
G
C
T
A
C
G
T
A
G
C
T
C
G
T
A
C
T
G
A
C
T
G
A
T
G
A
C
G
T
C
A
  

|  |  |
| --- | --- |
| p-value: | 1e-57 |
| log p-value: | -1.324e+02 |
| Information Content per bp: | 1.635 |
| Number of Target Sequences with motif | 126.0 |
| Percentage of Target Sequences with motif | 3.17% |
| Number of Background Sequences with motif | 161.2 |
| Percentage of Background Sequences with motif | 0.38% |
| Average Position of motif in Targets | 102.9 +/- 32.7bp |
| Average Position of motif in Background | 101.3 +/- 66.7bp |
| Strand Bias (log2 ratio + to - strand density) | -0.2 |
| Multiplicity (# of sites on avg that occur together) | 1.01 |
| Motif File: | file (matrix) reverse opposite |

### Similar de novo motifs found

|  |  |  |  |  |  |  |  |
| --- | --- | --- | --- | --- | --- | --- | --- |
| Rank | Match Score | Redundant Motif | P-value | log P-value | % of Targets | % of Background | Motif file |
| 1 | 0.961 | C T A G A G C T A C G T C A G T C T G A C G T A C G T A C T G A A T C G G C A T T C A G G C A T C T A G | 1e-54 | -125.070411 | 3.04% | 0.37% | motif file (matrix) |
| 2 | 0.857 | A G C T A C G T A C G T C G T A G T C A C G T A G T C A A C T G G C A T T A C G A C G T C A T G G A C T C T G A | 1e-49 | -114.576144 | 2.49% | 0.25% | motif file (matrix) |
| 3 | 0.789 | A G C T G C A T T C G A C G T A T C G A T C G A A T C G A C G T C A T G A G C T A T C G G C A T A C T G | 1e-22 | -52.329132 | 1.56% | 0.26% | motif file (matrix) |
